# Granulomatous Mastitis Mimicking Infection: A Case Report of Therapeutic Challenges and Evolving Management

**DOI:** 10.7759/cureus.111555

**Published:** 2026-06-26

**Authors:** Chuchu Zhao, Jie Huang, Tanveer Dhandwar, Ejike Oluka, Bahtiyar Toz

**Affiliations:** 1 Department of Medicine, NYC Health + Hospitals/Queens, Icahn School of Medicine at Mount Sinai, New York, USA; 2 Department of Pathology, NYC Health + Hospitals/Queens/Elmhurst, Mount Sinai Services, Icahn School of Medicine at Mount Sinai, New York, USA

**Keywords:** antibiotic nonresponse, breast abscess, granulomatous mastitis, idiopathic granulomatous mastitis, methotrexate

## Abstract

A 24-year-old woman presented with right breast pain and a progressively enlarging mass that had persisted for 4 months. It was initially managed as a breast abscess. However, after multiple courses of antibiotics, incision and drainage, and intralesional corticosteroid injections, her symptoms did not improve, and new areas of involvement developed. Breast ultrasound showed findings suspicious for an inflammatory lesion, and routine cultures remained negative. Breast core biopsy revealed non-caseating granulomatous inflammation, consistent with granulomatous mastitis. In the setting of a progressive course and poor response to prior therapies, systemic immunosuppressive treatment with methotrexate was initiated. At approximately 7 weeks after methotrexate initiation, she reported overall clinical improvement.

In this case, the lack of response to antibiotics raised concerns for an underlying inflammatory process, emphasizing the need for early biopsy and reassessment when the clinical course is atypical. It also reflects the clinical difficulty of deciding when to shift from antibiotics to anti-inflammatory or immunosuppressive treatment.

## Introduction

Granulomatous mastitis is an uncommon chronic inflammatory condition of the breast that most often affects women of reproductive age and frequently presents with breast pain, erythema, swelling, and mass-like lesions. Because these findings can resemble bacterial mastitis, breast abscess, and, less commonly, malignancy, diagnosis can be challenging, particularly early in the disease course [1,2].

Histologically, granulomatous mastitis is characterized by granulomatous inflammation, often with non-caseating granulomas, which reflect a granulomatous immune response but are not specific for idiopathic disease. The distinction between granulomatous mastitis and idiopathic granulomatous mastitis (IGM) is important. Granulomatous mastitis is a histologic pattern that may be associated with infection, systemic inflammatory disease, foreign-body reaction, malignancy-associated inflammation, or other secondary causes [1,2,3]. IGM is generally considered a diagnosis of exclusion after secondary causes have been reasonably evaluated [1,2,3].

In clinical practice, many patients are initially managed with antibiotics or surgical drainage because the presentation may mimic infection [2]. However, persistent or progressive disease despite antimicrobial therapy should prompt reassessment, tissue diagnosis, and consideration of an inflammatory process [3,4]. A major management challenge is determining when to transition from antimicrobial therapy to anti-inflammatory or immunosuppressive treatment while still acknowledging the limitations of negative cultures and the possibility of atypical infection. We describe the case of a young woman with presumed breast infection who did not respond to antibiotics and developed progressive disease, leading to reconsideration of the diagnosis and treatment strategy.

## Case presentation

A 24-year-old woman presented with a four-month history of right breast pain and a gradually enlarging mass. The symptoms initially presented as localized tenderness and redness around the areola and progressed over time to involve a large portion of the breast. She denied recent breastfeeding, trauma, fever, or systemic symptoms such as rash or joint pain. Laboratory evaluation showed no leukocytosis, with a white blood cell count of 7.60×10⁹/L (reference range 3.8×10⁹/L-10.5×10⁹/L) and CRP 7 mg/L (reference range ≤4 mg/L). Serum prolactin was normal at 12.6 ng/mL (reference range 3.4-24.1 ng/mL). Physical examination showed a large, irregular, tender mass involving multiple regions of the right breast, measuring approximately 4×4 cm on examination, with overlying hyperpigmentation and induration. There was no nipple retraction or discharge and no clinically significant axillary lymphadenopathy. Breast ultrasound revealed an ill-defined heterogeneous area with increased vascularity, consistent with an inflammatory process.

The patient was initially treated for a breast abscess. She was given amoxicillin-clavulanate 875-125 mg two times daily for 10 days and then switched to trimethoprim-sulfamethoxazole 800-160 mg two times daily for 10 days without improvement. Incision and drainage mostly produced bloody fluid with little purulence. Bacterial aerobic and anaerobic cultures showed no growth. Dedicated *Corynebacterium *culture, fungal culture, acid-fast bacilli culture, and molecular testing were not performed. Despite treatment, her symptoms persisted, with new areas of redness and firmness developing in different parts of the breast. Given the lack of clinical response to antibiotics, negative routine cultures, absence of systemic signs of infection, and repeat ultrasound showing no drainable abscess, an inflammatory etiology was considered. After clinical reassessment, she received two intralesional triamcinolone injections with only transient improvement in redness and pain, followed by continued progression of the breast mass. She was subsequently referred for rheumatological evaluation.

**Figure 1:**
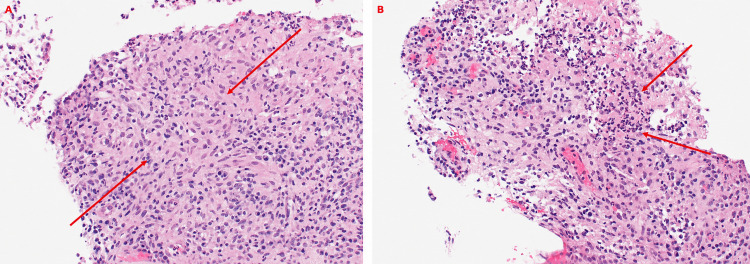
Histopathology of the right breast core biopsy (A) Hematoxylin and eosin staining shows non-caseating granulomatous inflammation with epithelioid histiocytes (arrows) in otherwise benign breast tissue. Original magnification ×20. (B) A separate field shows granulomatous inflammation with associated mixed inflammatory infiltrates, including neutrophil-rich areas and focal microabscess-like features (arrows), which may histologically overlap with cystic neutrophilic granulomatous mastitis. No evidence of malignancy was identified. Acid-fast bacilli and fungal stains were negative. Original magnification ×20.

Follow-up breast ultrasound after drainage and antibiotic therapy showed persistent inflammatory changes without significant improvement. No drainable abscess was identified. Breast core needle biopsy demonstrated non-caseating granulomatous inflammation in otherwise benign breast tissue, with no evidence of malignancy (Figure 1). Neutrophils and focal microabscess formation were present, creating histologic overlap with cystic neutrophilic granulomatous mastitis. Acid-fast bacilli and fungal stains were negative. Because these entities may overlap histologically, microbiologic correlation with cultures and/or PCR is important before diagnosing idiopathic granulomatous mastitis. In this case, cultures were negative, and the overall findings supported granulomatous mastitis after exclusion of malignancy and identifiable infection. Autoimmune testing was performed to exclude secondary causes of granulomatous inflammation. Serum IgG4 level was within the normal range, and autoimmune serologic testing, including antinuclear antibody, rheumatoid factor, anti-cyclic citrullinated peptide antibody, and antineutrophil cytoplasmic antibody, was negative.

With persistent progression, lack of response to antimicrobial therapy, and supportive biopsy findings, the diagnosis was revised to idiopathic granulomatous mastitis with an underlying inflammatory component. Methotrexate was initiated as the patient had extensive and progressive disease with a limited response to local corticosteroid therapy. Before starting methotrexate, counseling was provided regarding potential adverse effects, the need for laboratory monitoring, avoidance of pregnancy during treatment, and the importance of contraception because the patient was of childbearing age. Folic acid supplementation was prescribed.

At the most recent follow-up, approximately 7 weeks after methotrexate initiation, the patient reported overall clinical improvement, with decreased baseline breast pain and reduction in previously persistent lesions. Physical examination showed interval improvement in the right breast lesions, with mild residual tenderness and erythema in two areas. The follow-up plan included rheumatology follow-up every 3 months, monitoring of complete blood count and liver function tests, assessment of treatment response, and repeat breast imaging if symptoms persisted or progressed. Her diagnostic and therapeutic course is summarized in Table 1.

**Table 1:** Chronological clinical course and treatment timeline GMS: Grocott-Gomori’s methenamine silver stain; AFB: acid-fast bacilli stain

Approximate clinical interval	Clinical event	Findings and management
Initial presentation	Right breast pain and mass	Four-month history of right breast pain, redness, and enlarging mass without fever, recent breastfeeding, trauma, or systemic symptoms.
Early course	Empiric antibiotics	Treated sequentially with amoxicillin-clavulanate and trimethoprim-sulfamethoxazole for presumed infection, without sustained improvement.
1 month after initial antibiotic therapy	Incision and drainage	Mostly bloody drainage with minimal purulence was obtained. Culture showed no growth.
2-3 months after initial antibiotic therapy	Local corticosteroid therapy	Two right breast therapeutic corticosteroid injections produced only mild or transient improvement, with continued development of new areas of redness and mass-like changes.
4 months after initial antibiotic therapy	Repeat imaging	Repeat breast ultrasound showed persistent treated presumed granulomatous mastitis without significant interval change.
4 months after initial antibiotic therapy	Core biopsy	Breast core biopsy showed benign breast tissue with acute and focal non-caseating chronic granulomatous inflammation. GMS and AFB stains were negative.
5 months after initial antibiotic therapy	Methotrexate initiation	Methotrexate 15 mg weekly with folic acid 1 mg daily was started because of persistent disease despite antibiotics, drainage, and local corticosteroid therapy.
7 weeks after methotrexate initiation	Follow-up	The patient reported overall improvement. Repeat breast ultrasound and ongoing rheumatology follow-up were planned.

## Discussion

Granulomatous mastitis can be difficult to diagnose in practice, as it often presents with clinical and imaging features that resemble infection. It can also be challenging to manage, particularly when deciding when to shift from antimicrobial to anti-inflammatory or immunosuppressive therapy. In our case, the patient was initially managed as having an infectious process, with multiple antibiotic courses and drainage. However, the subsequent course was atypical, with persistent symptoms and new areas of involvement despite treatment, which led us to reconsider the diagnosis and management strategy.

Recent guidelines describe idiopathic granulomatous mastitis (IGM) as an inflammatory condition of the breast rather than a primary infection and recommend histological confirmation [1]. Granulomatous mastitis may be idiopathic or secondary to infection, systemic inflammatory disease, IgG4-related disease, sarcoidosis, foreign body reaction, or other causes; therefore, IGM should be diagnosed only after secondary causes have been reasonably excluded [1,2]. In this setting, empiric antibiotic therapy is generally not indicated when no pathogen is identified [1]. Despite this, antibiotics are still commonly used early in the course because the clinical presentation may closely resemble bacterial mastitis or abscess, which may delay biopsy and more appropriate treatment. Clinical experience and published data suggest that many patients respond to corticosteroids or other immunosuppressive therapies, supporting an immune-mediated process in selected cases [3,4].

However, negative microbiological studies do not completely exclude infection, particularly atypical organisms such as *Corynebacterium *species, mycobacteria, or fungi [1,5]. An important exception is cystic neutrophilic granulomatous mastitis (CNGM), which has been linked to Corynebacterium species and may require targeted antimicrobial therapy [1,5]. Because *Corynebacterium*-associated disease may be difficult to detect on routine culture, careful clinicopathologic correlation remains important [5]. In our case, aerobic and anaerobic bacterial cultures from incision and drainage showed no growth, and biopsy stains for acid-fast bacilli and fungal organisms were negative. However, dedicated *Corynebacterium *culture, fungal culture, acid-fast bacilli culture, and molecular testing were not performed. Therefore, although the overall clinicopathologic findings supported IGM after exclusion of malignancy and identifiable infection, a culture-negative atypical infectious trigger could not be completely excluded.

Histopathologic evaluation is essential to confirm granulomatous inflammation and to exclude malignancy and infectious mimics [1,2]. In this case, breast core needle biopsy demonstrated non-caseating granulomatous inflammation in otherwise benign breast tissue, with no evidence of malignancy. Neutrophils and focal microabscess-like features were present, creating histologic overlap with CNGM. These findings highlight why microbiologic correlation is important before diagnosing IGM. In our patient, the biopsy findings, negative available stains and routine cultures, negative autoimmune evaluation, normal serum IgG4 level, absence of systemic features, and persistent inflammatory course supported a diagnosis of IGM in the appropriate clinical context, while acknowledging the limitations of the infectious evaluation.

Management strategies vary and are usually guided by disease severity. A stepwise approach is often used, ranging from observation or non-steroidal anti-inflammatory drugs in milder cases to corticosteroids and immunosuppressive agents in more extensive or refractory disease [1]. Corticosteroids remain widely used and can be effective, although relapse is not uncommon, particularly in patients with persistent or extensive disease [4]. For patients who do not respond adequately, escalation to systemic immunosuppressive therapy is often considered. A network meta-analysis found that steroid-based combination regimens, particularly surgery combined with local and systemic corticosteroid therapy, ranked highest for reducing recurrence in idiopathic granulomatous mastitis [6]. Observational studies also suggest that agents such as methotrexate or azathioprine may help reduce recurrence, supporting a stepwise escalation strategy in selected patients [7].

In our patient, methotrexate was selected because of persistent and progressive disease despite multiple antimicrobial courses, incision and drainage, and only a limited response to intralesional corticosteroid therapy. The decision to use methotrexate was made after clinicopathologic correlation favored an inflammatory process, and counseling was provided regarding potential adverse effects, laboratory monitoring, and pregnancy avoidance. At approximately seven weeks after methotrexate initiation, the patient reported overall clinical improvement, with decreased baseline breast pain and reduction in previously persistent lesions. Examination also showed interval improvement, although mild residual tenderness and erythema remained in two areas. Therefore, this case suggests early partial clinical improvement after methotrexate initiation, but it cannot establish methotrexate efficacy or durability of response. Longer follow-up is needed to assess sustained disease control, recurrence, and the need for further therapy.

One area that remains unclear is the timing of treatment decisions. In practice, patients are frequently treated as having an infection initially, which can lead to repeated courses of antibiotics and a delay in escalation of therapy. Although current guidelines advise against empiric antibiotics in confirmed IGM, they offer limited guidance on when to change treatment [1]. As a result, management often depends on clinical response and ongoing reassessment rather than fixed timelines. In this case, the lack of sustained response to antibiotics, negative routine cultures, absence of systemic infectious features, no drainable abscess on follow-up imaging, and only transient improvement after intralesional corticosteroid injections supported escalation to systemic therapy. This case highlights the importance of reassessing the diagnosis when presumed infection follows an atypical or refractory course.

Granulomatous mastitis should be considered in patients with persistent or progressive breast inflammation and negative routine microbiological findings [3]. Recognizing this pattern early, obtaining tissue diagnosis, and balancing infection exclusion with timely anti-inflammatory or immunosuppressive therapy may help reduce unnecessary interventions and improve outcomes.

## Conclusions

Granulomatous mastitis should be considered in patients with persistent or progressive breast inflammation and negative microbiological findings, particularly when symptoms do not improve with antibiotics and drainage. Early biopsy can help establish the diagnosis and exclude malignancy. Negative cultures should be interpreted cautiously because atypical infections, including *Corynebacterium*-associated disease, may be difficult to confirm. In patients with progressive disease and limited response to initial therapy, escalation to corticosteroids or other immunosuppressive therapies may be considered, but treatment decisions should remain individualized and balanced against the uncertainty of infection exclusion and the limited evidence base. In this case, early partial improvement was observed after methotrexate initiation, but longer follow-up is needed to assess durability of response and recurrence.
